# Aquaporins in the lung

**DOI:** 10.1007/s00424-018-2232-y

**Published:** 2018-11-05

**Authors:** Oliver H. Wittekindt, Paul Dietl

**Affiliations:** Institute of General Physiology, Ulm University, Albert-Einstein-Allee 11, 89081 Ulm, Germany

**Keywords:** Lung, Aquaporin, Endothelium, Epithelium, Water transport

## Abstract

The lung is the interface between air and blood where the exchange of oxygen and carbon dioxide occurs. The surface liquid that is directly exposed to the gaseous compartment covers both conducting airways and respiratory zone and forms the air-liquid interface. The barrier that separates this lining fluid of the airways and alveoli from the extracellular compartment is the pulmonary epithelium. The volume of the lining fluid must be kept in a range that guarantees an appropriate gas exchange and other functions, such as mucociliary clearance. It is generally accepted that this is maintained by balancing resorptive and secretory fluid transport across the pulmonary epithelium. Whereas osmosis is considered as the exclusive principle of fluid transport in the airways, filtration may contribute to alveolar fluid accumulation under pathologic conditions. Aquaporins (AQP) facilitate water flux across cell membranes, and as such, they provide a transcellular route for water transport across epithelia. However, their contribution to near-isosmolar fluid conditions in the lung still remains elusive. Herein, we discuss the role of AQPs in the lung with regard to fluid homeostasis across the respiratory epithelium.

## Introduction

The pulmonary epithelium separates the aqueous interstitial compartment from the lining fluid of the conductive airways, of the respiratory airways and alveoli. This lining fluid, also denoted airway or alveolar surface liquid (ASL), has several functions: First, it shields the epithelial cells from gas exposure and thereby protects them from damage [84]. Second, it is the layer that contains surfactant [19], mucus [110], and a multitude of other constituents that enable proper lung function (mechanical properties, innate host defense, mucociliary clearance, etc.), which will not be further discussed here. The thickness and therefore the volume of this ASL is a critical parameter that needs to be kept in a volume range that guarantees an appropriate lung function [7].

ASL formation and stabilization both depend on fluid secretion and resorption, as a result of active ion transport. The sodium transport that drives water resorption in lung epithelia is predominantly limited by the epithelial sodium channel (ENaC) [35] whereas the Cl^−^ transport that drives fluid secretion is maintained by different anion channels, most importantly the cystic fibrosis transmembrane conductance regulator (CFTR) and Ca^2+^-activated Cl^−^ channels of the anoctamin group [75, 85]. Even though the major mechanisms and pathways for transepithelial ion transport across pulmonary epithelia are well established, the pathways for water flux remained elusive. This review focuses on the role of aquaporins in water transport across pulmonary epithelia.

## The protein family of aquaporins

When Macey and colleagues studied osmotically driven water flux across cell membranes of red blood cells [69], they observed that the plasma membrane acts as a sieve that enables bulk water flux. Membrane water permeability was sensitive to organomercury compounds. From these observations, it was concluded that water flux depends on water channels [16]. Later studies identified a 28 kDa channel forming integral membrane proteins (CHIP28) [80] that increases osmotic-driven water permeability in cell membranes when expressed in *Xenopus leavis* oocytes [81]. At that time, the γ-tonoplast intrinsic protein (γ-TIP) from *Arabidopsis thaliana* [3] and the water channel of the collecting duct (WCH-CD) [3] were identified as CHIP28-related proteins that form water channels in plasma membranes. Hence, water channels appeared to play a pivotal role in different species, and the new protein family was named aquaporins (AQP). They all share homology with the membrane intrinsic protein (MIP) superfamily, and the term aquaporin was introduced for those MIP homologs that form water-conducting pores [9]. Thirteen AQPs have been identified so far (Table 1). They share a common signature amino acid sequence composed by asparagines-proline-alanine, the so-called NPA box [43]. Recently, this protein family has been further subdivided into aquaporins that are specifically permeable for water and those that are permeable for water, glycerol, and urea, the so-called aqua-glycerol-porin glycerol facilitators [9]. AQP1 [2, 20, 116], AQP2 [116], AQP4 [30, 116], AQP5 [62, 116], and AQP8 [40, 55, 64] are denoted as aquaporins even though a study revealed that AQP1 also conducts glycerol [2]. Aqua-glycerol-porins are AQP3 [20, 116], AQP6 [33], AQP7 [40], AQP9 [41, 65], and AQP10 [42]. The novel subgroup of superaquaporins consists of AQP11 [37, 39, 71, 114, 115] and AQP12 [37]. AQP11 and AQP12 are distinct due to their low sequence homologies to the groups of aquaporins and aqua-glycerol-porins. They were formerly considered as AQPs with unusual NPA motives [38] that may form an AQP supergene family. Accordingly, they are named “superaquaporins” or “group III AQPs” [43].

**Table 1:** Superfamily of aquaporins. Subfamilies are given as AQP = aquaporin with selective water permeability, GlpF = aqua-glycerol-porins and facilitators, S-AQP = superaquaporins. Effects of aquaporin modulators pCMBS = para-cholormercuribenzenesulfonate, phloretin, Cu^2+^, and Hg^2+^ are assigned as − = inhibition, + = activation and ± 0 = no effect on permeability

AQP	Subfamily [9]	Permeability	pCMBS	Phloretin	Cu^2+^	Hg^2+^
AQP1	AQP	Water [1, 20, 116] Glycerol [1]	− [1, 2]		− [1, 2]	− [76]
AQP2	AQP	Water [116]	− [2]		± 0 [2]	− [59, 76]
AQP3	GlpF	Water [20, 116] Glycerol [20, 116] Urea [20]	− [20]	± 0 [20]	− [121]	− [59]
AQP4	AQP	Water [30, 116]				± 0 [30, 52]
AQP5	AQP	Water [62, 116]				− [62]
AQP6	GlpF	Water [33] Glycerol [33] Urea [33]				+ [33]
AQP7	GlpF	Water [40] Glycerol [40] Urea [40]				− [40]
AQP8	AQP	Water [40, 55, 64]				− [40, 64]
AQP9	GlpF	Water [41, 65] Urea [41, 65] Glycerol [65]				− [41, 65]
AQP10	GlpF	Water [42] Glycerol [42] Urea [42]		− [42]		− [42]
AQP11	S-AQP [37]	Water [71, 114, 115] Glycerol [71]				− [115]
AQP12	S-AQP [37]					

Most AQPs, namely AQP1–3, AQP5, and AQP7–11, are sensitive to HgCl_2_ [40–42, 59, 62, 64, 65, 76, 115]. The only mercury-insensitive AQP known so far is AQP4 [30, 52]. AQP6 was identified to be activated by mercury ions [33]. Copper inhibits AQP1 [1, 2] and AQP3 [1, 2, 121].

**Fig. 1:**
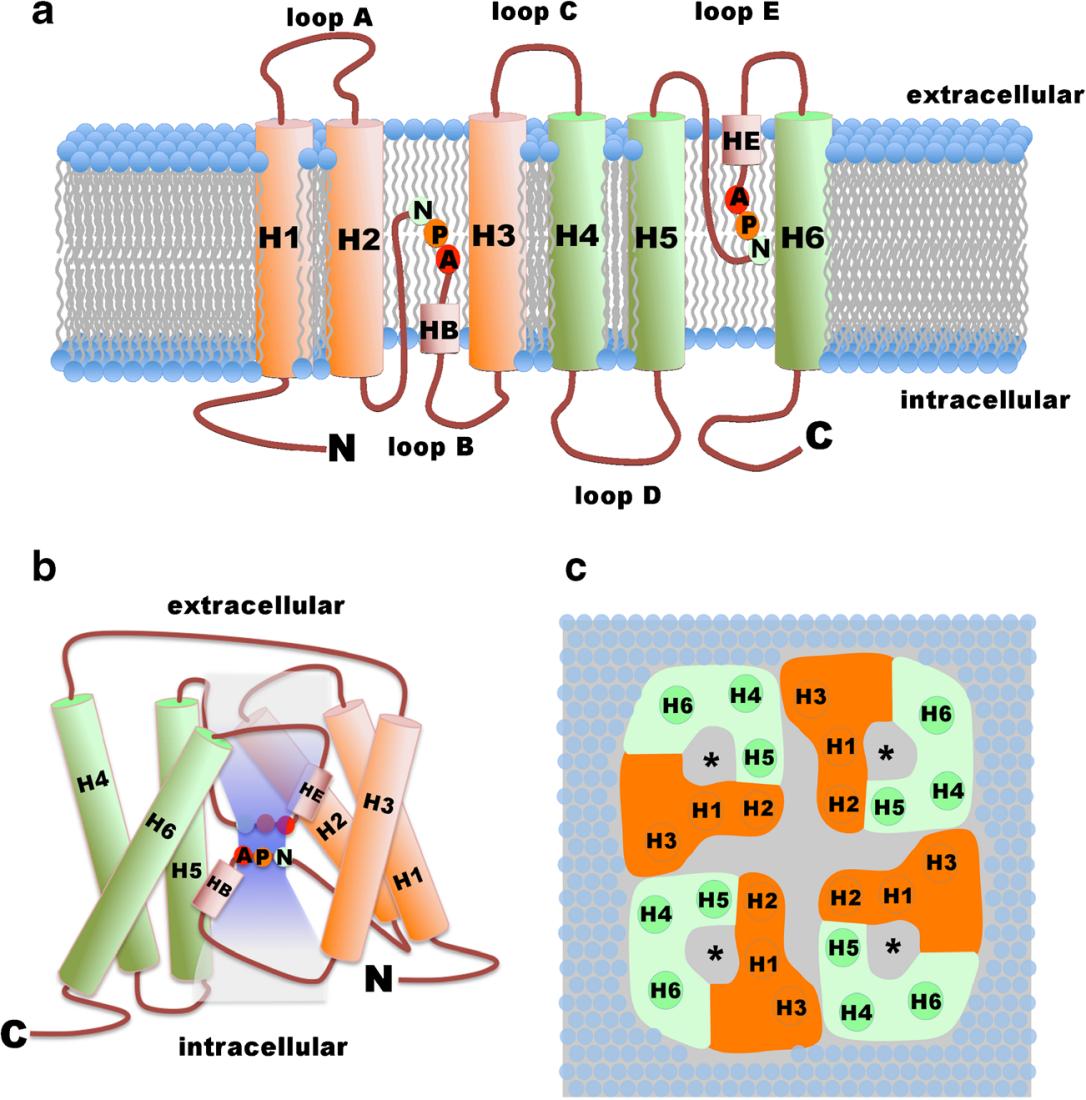
Schematic view of aquaporins. **a** Aquaporins contain cytosolic N- and C-terminal domains and six transmembrane spanning segments (H1 to H6). The extracellular loops (loops A, C, and E) link H1 with H2, H3 with H4, and H5 with H6. Intracellular loops (loops B and D) connect H2 with H3 and H5 with H6. The NPA motives (see text) are located in loop B N-terminal from the helix HB and in loop E N-terminal from the HE helix. **b** Schematic model of arrangement of the transmembrane spanning segments, the small pore helices HB and HE, as well as the NPA boxes around the water-conducting pore (shown in light blue). The obstruction of the center pore by the NPA motives according to the hour glass model is depicted. **c** View from above the extracellular surface on the top of an AQP tetramer. The arrangement of the transmembrane spanning segments H1 to H3 (given in orange) and H4 to H6 (given in green) is shown. Asterisks highlight the water pore of each AQP monomer. (All models were redrawn from [31, 56, 88, 100])

AQPs consist of six transmembrane spanning segments with cytosolic N- and C-terminal regions (Fig. 1a) with the first NPA box located in the “intracellular” loop region between the second and third transmembrane spanning segments (the so called B-loop) and the second NPA box situated within the “extracellular” loop between transmembrane segments 5 and 6 (named as E-loop). The water-permeable pore (Fig. 1b) is formed by a single AQP subunit via interaction of the B-loop and the E-loop that brings both NPA boxes in an anti-parallel orientation close to each other according to the so-called hourglass model [51]. This folding narrows the water pore and mediates the channel’s permselectivity [77, 87]. Even though each monomeric subunit forms its own water pore, AQP monomers are arranged as tetramers (Fig. 1c) in the plasma membrane [54, 106].

AQP activity in cells is highly regulated either by gating or by trafficking and modulation of AQP abundance in the plasma membrane. Modulation of AQP gating by phosphorylation of serine residues that localize at the inner pore region has been shown for plant AQPs [48] and AQP4 [120]. Changing electrostatic properties of the inner pore region seems to be a shared mechanism to modulate AQP gating in response to changes of the intracellular milieu such as cytoplasmic pH [4, 92, 101] or cytosolic Ca^2+^ concentration [4]. Beside channel gating, control of channel abundance at the plasma membrane is another mechanism to regulate AQP activity. This can be achieved either by changing AQP expression, trafficking towards and incorporation into the plasma membrane, or controlling the removal of AQP from the plasma membrane via ubiquitin-mediated mechanisms (reviewed for AQP2 in [50]).

## Aquaporins in the lung

Among the members of the AQP superfamily, only AQP1, AQP3, AQP4, and AQP5 are expressed in the lung. Their localization within the lung seems to be quite conserved between mice, rat, and human (summarized in Table 2). AQP1 is unequivocally detected in plasma membranes of endothelial cells that form the barrier of blood vessels along the airways as well as within the respiratory and alveolar regions [18, 21, 29, 44, 66, 72, 89, 102, 112]. AQP3 localizes to epithelial and basal cells of the nasal epithelium [58, 72], basal, and epithelial cells of the trachea, bronchi, and bronchioli [58, 72, 90]. Along the conducting and respiratory airways, AQP3 is incorporated into the lateral membranes of epithelial cells [90]. Also, cuboidal alveolar epithelial type II (ATII) cells express AQP3 [58, 90]. AQP4 was detected in basal, ciliated, and glandular cells of the nasal epithelium [18, 58, 72, 89, 118] and the trachea [72]. Along the bronchial epithelium, AQP4 localizes along the basolateral membrane of ciliated and intermediate cells [18, 58, 72, 118]. It was also detected in alveolar type I (AT-I) cells in the alveoli [18, 58, 89]. AQP5 was found in the apical membranes of epithelial cells and serous cells of gland acini of the nasal [58] bronchiolar epithelium [44, 58]. Along the more distal airways of the terminal and respiratory bronchioli, AQP5 is reported to be localized in the apical membrane of cuboidal cells [29, 44] and in AT-I cells of the alveolar epithelium [18, 21, 58].

**Table 2:** AQP localization in the lung

		Nasopharynx	Trachea	Bronchi	Bronchioli	Resp. airways	Alveolus	Endothelium
AQP1	Mice							[18, 29, 102]
	Rat							[21, 44, 66, 72, 89, 112]
	Human							[53]
AQP3	Mice			Epithelial cell abluminal side [18]				
	Rat		Basal cell membrane [89]	Basal cell membrane [89]	Cuboidal cells [89]	Cuboidal cells [89]		
	Human	Membrane of basal cells [58]	Lateral membrane of epithelial and basal cells [90]	Membrane of basal cells [58], lateral membrane of epithelial and basal cells [90]	Apical and basolateral membrane of columnar and basal cells [58], lateral membrane of epithelial and basal cells [90]	Cuboidal cells [58], lateral membrane of epithelial cells and basal cells [90]	AT-II cells [58], cuboidal cells of alveolar epithelium [90]	
AQP4	Mice			Epithelial abluminal side [18]			AT-I cells [18]	
	Rat			Neonatal lung [118]	[112]		AT-I cells [89]	
	Human	[58]		Columnar and intermediate cells [58]			[58]	
AQP5	Mice				Non-ciliated cuboidal cells, apical membrane [29]		[102] AT-I cells [18]	
	Rat			[44]	[44]		AT-I cells [21]	
	Human	Apical membrane of columnar and serous cells at gland acini [58]		Ciliated ducts and gland acini [58]			[58]	

## Barriers for fluid transport in the lung

Water that covers the surface of airways and alveoli originates in the vascular compartment, from where it travels through the interstitial compartment into the airspace. Accordingly, the water flux is a two-step process. First, water has to pass the endothelium that separates the vascular and interstitial compartment. This step can be described as a transendothelial water flux. Second, it has to move across the epithelium, called transepithelial water flux. The vascular endothelium and the epithelium both form barriers for the transendothelial/transepithelial water flux from the vascular compartment into the airways. Hence, when water travels from the vascular compartment onto the apical side of the lung epithelium, it has to pass two barriers in series, imposing an additive flow resistance.

The water permeability for a simple barrier can be quantified as the permeability coefficient for osmotically driven water flux across this barrier, according to [23] *P*_*f*_ = *v*_water_ × *J*_water_ / (*A*_surf_ × Δ_osm_) where *P*_*f*_ is the osmotic water permeability coefficient in (cm/s), *v*_water_ as the molar volume of water in (18 cm^3^/mol), *J*_water_ the water flux across the barrier in (mol/s), *A*_surf_ the surface area of the barrier in (cm^2^), and Δ_osm_ the osmotic gradient that drives water flux (osmol/cm^3^).

**Fig. 2:**
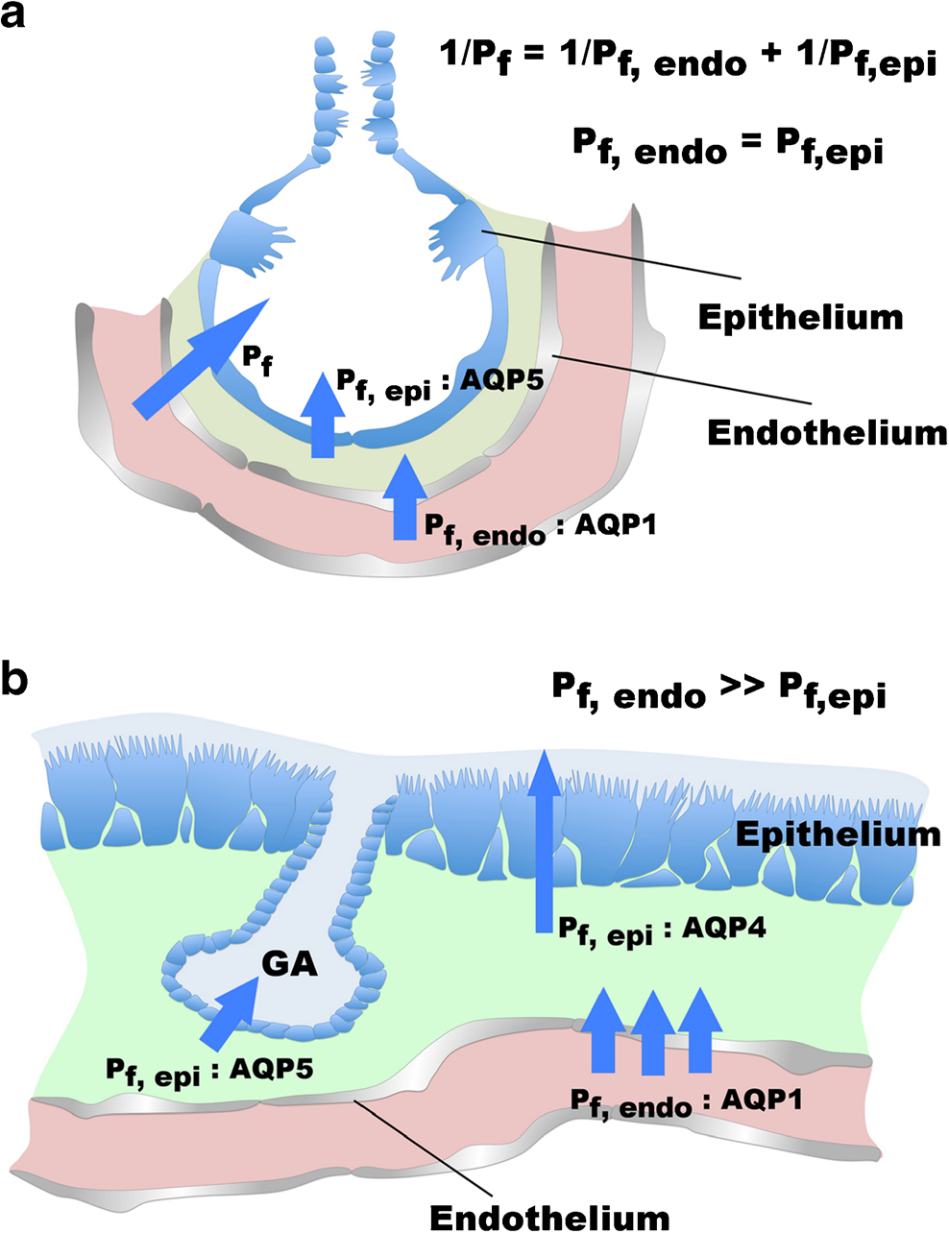
Barrier models for water transport in the lung. **a** Model for the respiratory segments. Endothelium and epithelium constitute barriers in series for water flux from the vascular compartment across the interstitium to the apical surface of the airways. Osmotic pressure-driven transendothelial/transepithelial water permeability (*P*_*f*_) depends on transendothelial (*P*_*f*,endo_) and on transepithelial (*P*_*f*,epi_) water permeability. *P*_*f*,endo_ and *P*_*f*,epi_ contribute equally to *P*_*f*_. AQP5 limits *P*_*f*,epi_, and AQP1 limits *P*_*f*,endo_. **b** Model of the conducting airways. Again, endothelium and epithelium act in series as barriers for water flux from the vascular compartment across the interstitium to the apical surface of the airways. In the airway segments, *P*_*f*,epi_ depends on AQP4 and is approximately a magnitude lower than *P*_*f*,endo_ that is limited by AQP1. Hence, *P*_*f*,epi_ dominates *P*_*f*_. GA marks glandular acini. Water permeability of the GA epithelium depends on AQP5

Transendothelial/transepithelial water permeability *P*_*f*_ for the respiratory airway segments [11] and the transendothelial osmotic water permeability *P*_*f*,endo_ of alveolar endothelial barrier [13] were quantified in perfused mouse lung models (Fig. 2a). Comparing both investigations reveals that alveolar transendothelial *P*_*f*,endo_ was twice as high as alveolar transendothelial/transepithelial *P*_*f*_ indicating that transendothelial and transepithelial water permeabilities contribute almost equally to transendothelial/transepithelial water permeability of the respiratory segments and the alveoli.

Investigations on dissected small airways [24] revealed (Fig. 2b) a transepithelial water permeability that is approximately a magnitude smaller than the transendothelial/transepithelial water permeability of the respiratory segments and alveoli [11]. This demonstrates that water permeability of the endothelial/epithelial barrier depends on its localization along the bronchial tree. It further suggests that proximal of the respiratory zone, the epithelium forms the limiting barrier for transendothelial/transepithelial water flux.

## Contribution of AQPs to transendothelial and transepithelial water permeability

Analyzing osmotically driven transendothelial/transepithelial water flux in intact sheep lung uncovered a route that depends on mercury-sensitive water channels [23]. This observation was the first functional proof for an AQP-mediated transendothelial/transepithelial water flux in the lung. The availability of AQP knockout mice enabled to identify those AQPs that limit water permeability in lung microvascular endothelium and lung epithelium. Studies in lungs of AQP knockout mice were performed on whole lungs, which makes it difficult to assess the contribution of AQPs to the osmotic water permeability of certain segments of the bronchial tree. Nonetheless, in whole lung experiments, the measured effects of AQP knockout can be mainly linked to the respiratory and alveolar segments simply because they make up by far the major part of the surface area across which the measured fluid flux occurs.

Knockout of AQP1 resulted in an approximately 10-fold decrease of transendothelial/transepithelial water permeability [6, 68, 95]. Dissecting transendothelial/transepithelial water permeability and microvascular transendothelial water permeability [6] revealed that AQP1 is the dominating route for water flux across the pulmonary microvascular endothelium. Furthermore, it underscores that water passes the endothelium predominantly via a transcellular route. This also applies to the human lung, where AQP1 null mutations decrease microvascular endothelial water permeability [53].

The transepithelial water flux along the respiratory segments is also affected by AQP5 knockout [68]. Again, the authors studied osmotic transendothelial/transepithelial water permeability and revealed that AQP5 knockout reduced water permeability by a factor of 10. This was further reduced 2- to 3-fold in AQP1/AQP5 double knockout animals. Even though transendothelial water permeability was not dissected from transendothelial/transepithelial permeability by direct measurements, they concluded that AQP5 mediates the dominating transcellular pathway across the alveolar epithelium.

AQP4 knockout reduced osmotic transendothelial/transepithelial water permeability only slightly [95]. Even though AQP4 is reported to be expressed in the alveolar epithelium [18], it does not contribute substantially to the water permeability of the epithelium along the respiratory segments. Investigations on dissected small airways [24] revealed that the osmotic transepithelial water permeability along these airway segments is HgCl_2_ insensitive [24]. Since AQP4 is known to be HgCl_2_ insensitive, it is likely that it contributes to the transepithelial water permeability of the conductive airway segments more than it does for the alveolar barrier and the respiratory segments.

Taken together, all these experiments unequivocally demonstrate that water travels across the lung microvascular endothelium as well as across the lung epithelia via an AQP-dependent transcellular pathway rather than along a paracellular route across tight junctions.

## AQPs and hydrostatic pressure-driven water permeability

Beside osmotic gradients, hydrostatic pressure gradients across endothelial/epithelial barrier can also force water to move from the vascular lumen onto the apical surface of the lung epithelium. Raising the hydrostatic pressure in pulmonary capillaries causes lung edema by forcing the filtration of fluid from the vascular into the interstitial compartment. When capillary pressures exceed a certain threshold, additional fluid movement across the epithelium into the alveolar space may occur. An increase of the intravascular hydrostatic pressure affects water flux across the endothelial/epithelial barrier of the lung first by modulating endothelial permeability directly [119]. Secondly, this facilitates transepithelial water transport via activating active ion secretion [93]. Therefore, conclusions about the water permeability derived from hydrostatic pressure-driven water fluxes must be interpreted with caution. Although AQPs contribute to hydrostatic-driven transendothelial and transepithelial water flux, their contribution may be different from osmotically driven water fluxes.

The role of AQP in hydrostatic edema formation was investigated by increasing the hydrostatic pressure in the pulmonary artery of perfused lungs from AQP knockout mice [6, 95]. The contribution of AQP1 to hydrostatic-driven fluid accumulation was investigated in two different experimental setups. The first one investigated a situation in which the intravascular hydrostatic pressure was adjusted simply by modulating the intravascular pressure of the pulmonary artery while keeping the hydrostatic intravascular pressure in the pulmonary vein at 0 cm H_2_O. In this setting, AQP1 knockout reduced hydrostatic-driven water accumulation more than 2-fold [6]. In another setting, the hydrostatic intravascular pressure of the pulmonary artery was adjusted while the venous outflow pressure was kept at 5 cm H_2_O. In this setting, AQP1 knockout reduced pulmonary fluid accumulation by a factor of 1.4 only [95]. This reflects the pathophysiological situation during hydrostatic edema formation and convincingly demonstrates the importance of venous outflow pressure on lung edema formation. The small effect of AQP1 knockout might be explained by the fact that venous outflow pressure increases vascular endothelial water permeability in an AQP-independent manner [95, 119].

Contrary to AQP1 knockout, knockout of AQP5 had no effect on hydrostatic pressure-driven fluid accumulation in the lung [68]. AQP5 is localized in the epithelium [18, 29, 102] where it mediates a transcellular water permeability [68]. Hence, AQP5 is not involved in transendothelial water flux that is driven by increases in intravascular hydrostatic pressure and causes lung fluid accumulation in the interstitium.

All these experiments unequivocally revealed that AQP knockout has a considerably lower effect on hydrostatic pressure-driven transendothelial water flux than it has on the osmotic-driven one. AQPs form pores that selectively allow the solvent (water) to permeate endothelial and epithelial barriers but not osmotically active solutes. Hence, the hydrostatic pressure-driven water flux establishes an osmotic gradient across the barriers that counteracts the hydrostatic pressure gradient. This is essentially a “safety mechanism” that effectively prevents edema formation.

## AQP5 in submucosal glands

Beside its effect on *P*_*f*_ in the alveolar epithelium, AQP5 plays a pivotal role in mucus secretion of submucosal glands (Fig. 2b), where it is localized in the apical membrane of serous acinar cells [58]. Active sodium chloride secretion drives the water transport into the gland’s lumen. The gland’s acini and serous tubules harbor mucous cells, which produce and secret glycoproteins via exocytosis. Once secreted into the glands’ lumen, the glycoproteins form a viscous hydrogel that is transported along the glandular duct onto the airway surface. Knockout of AQP5 reduces fluid secretion by approximately 60% [94]. Furthermore, AQP5 knockout impairs the solute composition of the exudate since it results in elevated protein and Cl^−^ concentrations [94]. This underscores that ion and protein secretion remains unaffected by AQP5 knockout, whereas the water secretion into glands’ acini and serous tubules was reduced.

## Transepithelial transport models, AQP knockout, and lung fluid homeostasis

Despite their impact on osmotically driven water permeability of endothelial and epithelial barrier in the lung, AQPs seem to affect lung fluid homeostasis only barely. Single knockout for AQP1 [95, 97], AQP3 [125], AQP4 [95, 96], and AQP5 [68, 94, 96] as well as double knockout for AQP1/AQP4 [95], AQP1/AQP5 [96, 97], and AQP3/AQP4 [97] were intensely investigated. However, these experiments did not uncover any impairment of ASL volume homeostasis, ionic ASL composition, and humidification of inhaled air at normal, quasi-physiological, conditions. The most surprising observation was that none of the AQP knockouts impaired near-isotonic fluid transport across the pulmonary epithelium, at least not in mice. There is no evidence that this lack of effect could be due to compensating changes in ion transport across the epithelium, such as altered ion channel and/or Na^+^/K^+^ ATPase activity in AQP knockout. Even though such compensating mechanisms of AQP knockout on water transport cannot be completely excluded, changes of ion transport-driven water transport in AQP knockout vs wild type animals were not detected, neither after maximal activation of ion transport [6, 68, 96] nor due to changes in ion composition of ASL [97].

**Fig. 3:**
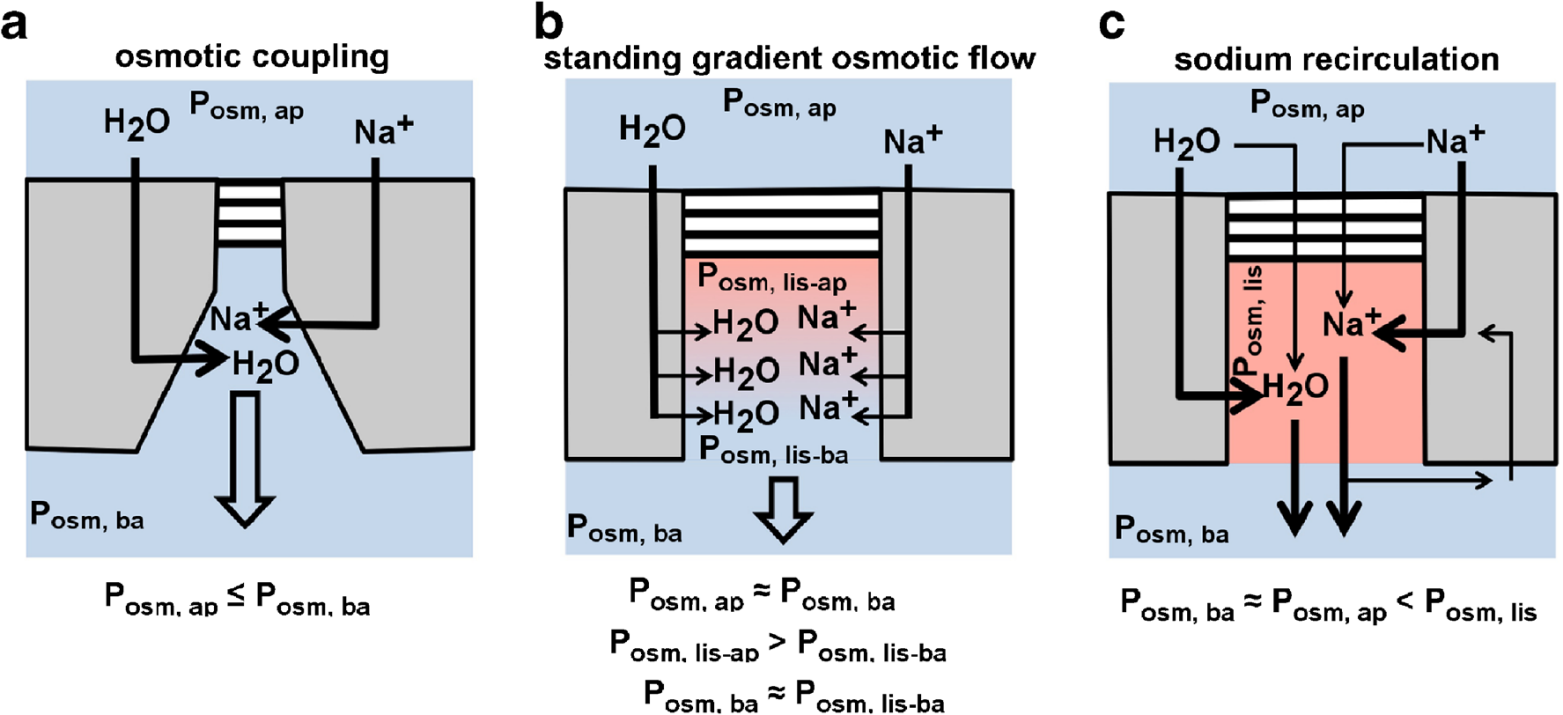
Comparison of osmotic coupling, standing gradient osmotic flow, and sodium recirculation models for near-isotonic water transport. Colors of extracellular compartments encode for their osmolality: red > blue. **a** Osmotic coupling model: Water and solutes travel via a transcellular route only. Apical osmolality (*P*_osm,ap_) almost equals basal osmolality (*P*_osm,ba_). The model distinguishes the apical, the basolateral, and the intracellular compartment. **b** Standing gradient osmotic flow model: Solutes and water travel via a transcellular route. Solutes that enter the lateral intercellular space establish an osmotic gradient. Within this gradient, osmolality drops from the apical pole of the lateral intercellular space (*P*_osm,lis-ap_) towards its basal pole (*P*_osm,lis-ba_). *P*_osm,lis-ba_ is almost equal to the osmolality of the basal compartment (*P*_osm,ba_). This model distinguishes the lateral interstitial space from the apical, the basal, and the intracellular compartment. **c** Sodium recirculation model: Water and solutes travel via a transcellular and via a paracellular route. Solutes that enter the lateral interstitial space increase its osmolality (*P*_osm,lis_). Osmolality of the resorbed fluid that exits the lateral intercellular space is adjusted to osmolality of the basal compartment (*P*_osm,ba_) by Na^+^ uptake across the basal cell membrane (Na^+^ recirculation). *P*_osm,ap_ is almost equal to *P*_osm,ba_. *P*_osm,lis_ is larger than *P*_osm,ap_ and *P*_osm,ba_. This model distinguishes the apical, the basal, the intracellular compartment, and the lateral intercellular space

One of the earliest models of transepithelial electrolyte and water transport is the “osmotic coupling model” [14]. This model assumes that ions are pumped across the epithelium and hence establish an osmotic gradient, and water follows subsequently by osmosis. Near-isotonicity of water transport is achieved simply by osmotic water permeability that is sufficiently high to achieve quasi-isotonic fluid transport (Fig. 3a). This model was refined by the so-called standing gradient osmotic flow model (Fig. 3b) that considers the lateral interstitial or lateral intercellular space as an additional and independent coupling compartment into which solutes and water are transported [108]. The transport into the lateral interstitial space establishes an osmotic gradient, which drops from the closed side (the apical mouth of the lateral space that is sealed by tight junctions, *P*_osm,lis-ap_) towards the open mouth at the basal side (*P*_osm,lis-ser_). According to this model, the gradient and hence the osmolality of the emergent fluid depend on (i) the length of the lateral space, (ii) the amount of transported ions, and (iii) the osmotic water permeability of the lateral membranes. Another refinement of the osmotic coupling model is the so-called Na^+^ recirculation model (Fig. 3c) [60, 104]. It also takes the lateral intercellular space as a coupling compartment for water and solute transport into account. In addition to the abovementioned models, the “Na^+^ recirculation model” considers also solutes and solvent that enters the lateral intercellular space from the apical side via the tight junctions. The Na^+^ that escapes from the lateral intercellular space at its basal side is partly guided back into the cell. This “recirculation” of Na^+^ across the basal membrane reduces the osmolality of resorbed fluid to reach near-isotonicity. [60]. The Na^+^ recirculation model considers the lateral intercellular space as a coupling compartment of homogenous composition. Again the lateral water flux needs water pores that provide an osmotic water permeability to the membranes [60, 61]. Within their limitations, all these models are applicable to near-isotonic water transport across cellular barriers. However, they all contradict the observation that the lack of AQPs does not affect transepithelial water transport in mouse lung.

Former hypotheses focused on the fact that lung epithelia have a considerably low resorption rate that presumably does not need a high osmotic water permeability of adjacent membranes [68]. MacLaren and colleagues introduced what they called a “basic compartment model” [70]. This model considers a transcellular-only transport and a coupling compartment that presumes the lateral intercellular space and the serous compartment as one compartment with a homogenous composition. This simple model reflects the AQP knockout effects surprisingly well. It revealed a non-linear dependence between water transport and osmotic permeability and thereby predicts that epithelial water transport becomes independent from AQPs when its overall water permeability is at least two magnitudes higher than its solute transport rate (The authors assumed that AQP knockout reduces water permeability by approximately 90% [70]). This estimation agrees with measured reductions of osmotic water permeability in AQP−/− mice [6, 68, 94–96]. Furthermore, this model features that the osmotic gradient between apical and coupling compartment decreases when osmotic pressure-driven water permeability increases, a feature that is shared also by the “standing gradient osmotic flow model” [73]. Apparently, the large osmotic pressure-driven water permeability in conjunction with almost isosmolar conditions explains the independence of water transport from AQPs in lung epithelia.

We recently found that human bronchial epithelia cultivated at air liquid interface conditions switch from a low resorptive state into a high resorptive state, when epithelia were overlaid with isotonic liquid. This switch happened several hours after increasing the apical surface liquid volume and coincided with the incorporation of AQP3 into the lateral cell membranes and a resulting increase in osmotic pressure-driven water permeability [90]. Obviously, lung epithelia adjust their water permeability to compensate for sustained increases in apical surface liquid volumes. This gives rise to the hypothesis that under conditions of an apical volume load, AQP3 is needed to increase water resorption and that AQPs are indeed important for fluid transport. This conclusion appears somehow contradictory to the results obtained from the experiments on AQP knockout mice. However, a direct transfer of results from mice to human lung epithelia is difficult for a couple of reasons:

1. Human bronchial epithelia and mouse lung epithelia differ strongly in their cellular composition. Compared to humans, mice show a reduced fraction of basal cells and an enlarged fraction of club cells in epithelia along trachea and main bronchi [36]. Possibly these differences in cellular composition may explain the contradictory results on AQP dependency of water transport in human and mouse lung epithelia.
2. The only model that explains the independence of water transport across airway epithelia from AQP expression and osmotic-driven water permeability, the basic compartment model [70], is based on the assumption that the lateral intercellular space and the basal compartment form one compartment of homogenous composition. This requires a non-limited exchange of water and solutes between these compartments. In pseudo-stratified epithelia, basal cells modify the lateral intercellular space at its basal mouth and may form a barrier that limits molecule exchange between lateral intercellular space and basal compartment. In this respect, namely in the amount of basal cells, human and mouse epithelia differ from each other. Therefore, the simple compartment model likely does not apply to the human epithelium as well as it does to murine one.
3. The geometry of epithelial cells differs between human and mouse lung [36]. The epithelia in mice comprise epithelial cells with a more isoprismatic shape, whereas human upper airway epithelia are formed by high prismatic cells. Hence, the geometry of the lateral intercellular space in mice lung epithelia meets more the criteria for the coupling compartment as presumed by the basic compartment model [70]. The geometry of the lateral intercellular compartment of the human upper airway epithelia corresponds more to the one described by the standing gradient model [108]. According to the standing gradient model, the lateral intercellular space does not form an isotonic but a hypertonic coupling compartment, especially when ion transport into this space is elevated, e.g., when apical surface liquid volumes are expanded. Under these conditions, the water transport rate would become dependent from AQPs.

Large osmotic gradients between adjacent compartments depend on active transepithelial ion transport, first to establish the gradient and second to maintain it by counteracting the back leak of ions. This active transport is energy consuming and increases in proportion to the osmotic gradient [8, 49]. Near-isosmolal transport that is guaranteed by a sufficiently high AQP water permeability minimizes the energy consumption that is needed for appropriate water transport. This aspect is of importance especially in the lung, where a constant water transport across a large surface area is needed to maintain air-liquid interface conditions.

## Role of AQPs in the lung under special conditions and disease

The results from AQP knockout animals led to the conclusion that AQPs do not play a major role in lung fluid homeostasis. Neither a significant lung phenotype nor an impaired lung growth during fetal and/or perinatal stages could be detected in the knockout animals. The reason for this lack still remains unclear. However, there is emerging evidence from studies on lung diseases that AQPs are involved especially in those lung diseases that are caused or at least accompanied by perturbed airway surface liquid volume homeostasis like asthma, chronic obstructive pulmonary disease (COPD), and acute lung injury. AQPs appear also to be important during late embryonic and perinatal stages of lung development and maturation as well as lung aging.

### AQP during lung development and aging

Fluid movement across the pulmonary epithelium is developmentally regulated. During fetal life, water secretion into premature alveolar spaces and airways is needed for lung growth [10, 15]. At birth, water transport has to switch from a more secretory to more resorptive transport phenotype. This switch establishes the organo-specific air-liquid interface and triggers the change form placental to alveolar gas exchange. Indeed, early studies already identified changes in AQP expression levels during perinatal stages. In the rat lung, AQP1 is already expressed during early stages in fetal life. Its expression levels rise shortly before birth and the first weeks after birth and adulthood [103, 117, 124]. AQP4 expression is fairly low during prenatal stages and increases transiently within first days after birth [103, 117, 124]. Finally, AQP5 expression rises perinatally and then steadily increases until adulthood [103, 117, 124]. The perinatal change in AQP expression patterns is paralleled by an increase in epithelial osmotic-driven water permeability that depends on increased water channel activity [12]. Glucocorticoids and catecholamines (β-receptors) are both proposed factors that facilitate perinatal water clearance in the lung and accelerate specifically AQP4 upregulation at birth [118]. Another factor that triggers AQP4 upregulation seems to be the elevation of the O_2_-partial pressure when airways are filled with air [86]. During aging, AQP1 and AQP5 expression levels decrease again, and as a consequence also the osmotic-driven water transport between capillary and airways [122].

These results underscore that AQP expression renders water permeability and therefore play an important role in adjusting water transport and airway liquid homeostasis to specific needs at certain developmental and age-dependent stages in the lung.

### AQP and acute lung injury

Characteristic clinical features of an acute lung injury are a diffuse bilateral alveolar damage and an acute exudative phase. Alveolar edema formation occurs especially during the exudative phase and goes along with an increase in alveolar fluid and airway surface liquid volume accumulation. The volume increase of water in the air-filled compartment has to be counteracted by increased fluid resorption that is considered to be supported by an increase of AQP-mediated osmotic permeability of the epithelium [79]. As outlined above, an apical surface liquid volume load triggers AQP3 to incorporate into lateral membranes of primary cultivated human bronchial epithelia [90]. Thereby, the osmotic water permeability of the epithelium increases, and it switches into a high resorptive state, during which the excessed fluid is resorbed [90]. This could be another possible safety mechanism that counteracts alveolar edema formation and clearly indicates possible roles of AQPs in lung injury. Indeed, patients with acute lung injury and diffuse alveolar damage exhibit increased expression levels for AQP1 and AQP5 but not for AQP1 [79].

Possible roles of AQP in lung injury, acute respiratory distress syndrome, lung edema formation, and clearance were intensively investigated in animal models. In these models, several agents were used to introduce lung injury, and one should consider that they target different lung structures and thereby could variably influence the course of lung injury. Agents like oleic acid and bacterial exotoxins bind to plasma proteins when given intravenously, and hence, they were considered to target primarily the vascular endothelium [74]. They can be distinguished from those that act primarily on the epithelium and that were usually instilled intratracheal-like acid (aspiration) or bleomycin [74]. Mechanical ventilation and hyperoxia are also considered to act predominantly on the epithelium [74]. In addition, treatments like ischemia/reperfusion or sepsis caused by intravenous administration of bacteria or cecal ligation and puncture target both the epithelium and the endothelium [74].

Even though each model causes lung injury in different and model-specific ways, studies that systematically address model-specific effects on lung AQPs are rare. In mice, it seems that changes in lung AQP expression depend on the agent used to induce lung injury [105]. Intratracheal administration of LPS causes AQP5 downregulation whereas ventilator-induced lung injury and intratracheal HCl instillation decrease AQP4 expression levels [105]. Even though the authors of this study discriminated between effects on the endothelium or epithelium, respectively, one should consider that all compounds were given intratracheally, and hence, all compounds targeted the epithelium first before they accessed the endothelium. In rat models, LPS reduced expression levels for AQP5 as well as AQP1 [27, 29, 34, 44, 45, 47, 91, 99, 111], while AQP3 and AQP4 seemed to remain unaffected [91]. There was no difference between intratracheal versus intravenous LPS administration obvious from these studies.

Ventilator-induced lung injury (VILI) targets predominantly the epithelium, and it was recognized that HgCl_2_ worsened VILI-induced water accumulation in lungs. This effect was attributed to the blockage of AQP1 and AQP5 [26]. In rat lungs, VILI reduced expression levels of AQP1 [22, 46], whereas AQP5 expression remained unaffected [22]. A more recent study revealed that AQP5 expression increases gradually with the duration of mechanical ventilation with low tidal volumes [21]. In these experiments, no edema formation was measured. These observations can be considered as evidence for a protective role of AQPs against VILI.

### Expression of AQPs changes also in other less frequent elaborated lung injury models

Hypoxia downregulates AQP1 expression [63, 112]. Ischemia reperfusion of lung tissue increased AQP1 expression, and AQP1 knockout severs lung injury and impairs its resolution after ischemia reperfusion [25]. Another study showed that ischemia-reperfusion of the lung reduces AQP5 expression [113]. Acute kidney injury often precedes and/or causes lung injury [17]. Indeed, ischemia reperfusion of the kidneys results in lung injury in rat that is also accompanied by reduced AQP5 expression in lung tissue [82]. A latter study revealed also changes in AQP1 expression levels in rat lung after kidney injury-induced lung injury [67].

These studies clearly show that lung injury changes pulmonary AQP expression regardless of its cause, most frequently the expression of AQP1 and AQP5. However, it is not yet clear if the observed changes play a causal role for the generation and course of lung injury. A protective role of AQPs against lung injury during the resolving phase seems likely.

### AQP in COPD

COPD is one of the leading causes of death worldwide. Its hallmarks are an increased resistance of the small airways and emphysematous remodeling of lung tissues. Remodeling of lung tissues is associated with a chronic innate and adaptive immune response that is paralleled by mucus hypersecretion and impaired mucus hydration [32]. There is a correlation of decreased AQP5 expression and protein levels with COPD severity [107, 124]. In addition, polymorphisms in the AQP5 gen are associated with a decline of lung function in COPD patients [28, 78]. AQP5 localizes in the apical membrane of serous cells at bronchial gland acini [58], where it facilitates fluid secretion [94]. Submocosal glands of AQP5 knockout mice still produce an exudate. However, the produced exudate is enriched for proteins, reflecting an reduced mucus hydration in AQP5−/− animals [94]. One has to consider that AQP5−/− mice do not suffer from impaired alveolar fluid clearance [96], and hence, it is unlikely that an impaired transepithelial fluid transport causes reduced mucus hydration in AQP5−/− mice. The reason for the observed mucus hypohydration might be the impaired exocrine function of the mucus producing glands that results in enriched mucus content of the exudate. In combination with an inflammation-induced mucus hypersecretion, this may lead to mucus enrichment with increased viscosity and facilitate mucus plaque formation and finally airway obstruction.

### AQP and asthma

Asthma is an inflammatory process of the airways. On the basis of the underlying innate and adaptive immune reactions, asthma can be subdivided into two major endotypes: T-helper type 2 cells high (T_H_2-high) and T-helper type 2 cells low (T_H_2-low) endotype [98]. The T_H_2-high endotype is characterized by eosinophil accumulation whereas T_H_2-low endotype can be identified by elevated levels of neutrophils.

In a recent study, increases in AQP1 and AQP5 content in the sputum were proposed as possible diagnostic markers for mild asthma [123]. Although AQP levels in BALF are not used as diagnostic markers so far, this study points to a potential role of AQPs in asthma.

In mouse models, asthma—and especially allergic asthma—are induced by two different treatments: ovalbumin and IL-13 treatment. Both ovalbumin and intratracheal instillations of IL-13 [109] induce an intense allergic airway inflammation in mouse lungs [5]. Both treatments result in asthma symptoms. However, asthma induced by IL-13 is closely related to the T_H_2-high asthma endotype that is driven by elevated IL-13 levels.

Asthma models based on ovalbumin instillation exhibit changes in expression levels of AQP1, AQP3, AQP4, and AQP5. However, AQP protein abundance does not mirror mRNA expression. AQP1, AQP4, and AQP5 mRNA levels were reduced in ovalbumin-challenged mouse lungs, whereas AQP3 mRNA remained unaffected [57]. The same study revealed reduced protein abundance for AQP1, AQP3, and AQP5 with strongest reduction for AQP3 protein abundance. AQP4 protein levels were observed to be elevated [57]. Another study on ovalbumin-induced asthma models also reported reduced mRNA levels for AQP1, AQP4, and AQP5 but elevated AQP3 mRNA levels [18]. IL-13-induced asthma resulted in changes of lung AQP expression pattern that differed from the one obtained after ovalbumin treatment. IL-13 transiently upregulated AQP3 and downregulated AQP5 mRNA levels. The strongest change in mRNA levels was observed on the first day after intertracheal IL-13 instillation for both AQP3 and AQP5 mRNA. In these experiments, changes in protein abundance mirror the change in mRNA levels [57]. Interestingly, anti-asthmatic agents alleviate ovalbumin-induced changes in AQP1 and AQP5 mRNA expression and protein abundance [18, 83]. These results unequivocally underscore that AQP function is affected in lung tissue of asthma patients. Nevertheless, it is not yet elaborated to which extent these changes contribute to the course of the disease.

## Conclusion and future perspective

There is a huge body of evidence that AQPs play a pivotal role for water transport in the lung. AQP expression changes during perinatal stages of lung maturation when the epithelium switches from a secretory to a resorptive phenotype. AQP expression is also disturbed in several lung diseases that are accompanied by impaired water transport function such as lung injury, asthma, or COPD. Contrary to that, AQP knockout did not reduce the water resorption rate in the lung, although AQP knockout reduced osmotic water permeability by roughly 90%. The results from the AQP knockout experiments were explained by two conditions that are specific for the pulmonary epithelium: (i) near-isotonic conditions and (ii) high osmotic water permeability in relation to a fairly low ion transport rate. As long as these conditions apply to the pulmonary epithelium, water transport remains almost independent from AQP activity. When the transport conditions deviate from near-isotonicity, as it is the case for fluid secretion in submucosal glands, or when the ion transport rate increases over longer time periods, as it is observed in primary cultivated human bronchial epithelia after an apical volume load, osmotic water permeability matters. This would explain the observed changes in AQP expression and activity during perinatal stages of lung maturation as well as during lung diseases with impaired pulmonary fluid clearance. In these cases, the change in AQP expression adjusts water resorption to match the need or to counteract non-adequate airway surface liquid volumes. Beside these compensatory or protective functions, a change in AQP expression might also sever or even cause lung fluid accumulation.

To further understand the roles of AQPs in the lung, we need a more detailed analysis of lung epithelial transport. From a mere investigation of ion transport rates, it is not possible to conclude fluid transport rates when near-isosmotic conditions do not apply any more. In this case, water transport needs to be measured directly. Furthermore, the contribution of the tight junction for water and electrolyte transport is far from completely understood. Finally, the coupling compartment, which is doubtlessly important for water transport, requires further investigation, despite its difficult experimental and technical accessibility.

Even when these basic issues are better understood, it will still be a great challenge to elucidate the principles involved in the regulation of AQP expression and function in the lung.
